# Characterization of Laminins in Healthy Human Aortic Valves and a Modified Decellularized Rat Scaffold

**DOI:** 10.1089/biores.2020.0018

**Published:** 2020-12-07

**Authors:** Carl Granath, Hunter Noren, Hanna Björck, Nancy Simon, Kim Olesen, Sergey Rodin, Karl-Henrik Grinnemo, Cecilia Österholm

**Affiliations:** ^1^ Division of Cardiothoracic Surgery, Department of Molecular Medicine and Surgery, Karolinska Institutet, Stockholm, Sweden.; ^2^ Cell Therapy Institute, Dr. Kiran C. Patel College of Allopathic Medicine, Nova Southeastern University, Davie, Florida, USA.; ^3^ Cardiovascular Medicine Unit, Department of Medicine, Center for Molecular Medicine, Karolinska Institutet, Karolinska University Hospital, Stockholm, Sweden.; ^4^ Department of Bioscience, University of Skövde, Skövde, Sweden.; ^5^ Department of Chemistry, Ångström Laboratory, Uppsala University, Uppsala, Sweden.; ^6^ Chemistry I, Department of Medical Biochemistry and Biophysics, Karolinska Institutet, Stockholm, Sweden.; ^7^ Division of Cardiothoracic Surgery and Anesthesiology, Department of Surgical Sciences, Uppsala University, Akademiska University Hospital, Uppsala, Sweden.; ^8^ Division of Clinical Genetics, Department of Molecular Medicine and Surgery, Karolinska Institutet, Stockholm, Sweden.

**Keywords:** extracellular matrix, heart valves, laminin, tissue engineering

## Abstract

Aortic valve stenosis is one of the most common cardiovascular diseases in western countries and can only be treated by replacement with a prosthetic valve. Tissue engineering is an emerging and promising treatment option, but in-depth knowledge about the microstructure of native heart valves is lacking, making the development of tissue-engineered heart valves challenging. Specifically, the basement membrane (BM) of heart valves remains incompletely characterized, and decellularization protocols that preserve BM components are necessary to advance the field. This study aims to characterize laminin isoforms expressed in healthy human aortic valves and establish a small animal decellularized aortic valve scaffold for future studies of the BM in tissue engineering. Laminin isoforms were assessed by immunohistochemistry with antibodies specific for individual α, β, and γ chains. The results indicated that LN-411, LN-421, LN-511, and LN-521 are expressed in human aortic valves (*n* = 3), forming a continuous monolayer in the endothelial BM, whereas sparsely found in the interstitium. Similar results were seen in rat aortic valves (*n* = 3). Retention of laminin and other BM components, concomitantly with effective removal of cells and residual DNA, was achieved through 3 h exposure to 1% sodium dodecyl sulfate and 30 min exposure to 1% Triton X-100, followed by nuclease processing in rat aortic valves (*n* = 3). Our results provide crucial data on the microenvironment of valvular cells relevant for research in both tissue engineering and heart valve biology. We also describe a decellularized rat aortic valve scaffold useful for mechanistic studies on the role of the BM in heart valve regeneration.

## Introduction

Aortic valve stenosis is one of the most prevalent cardiovascular disorders in the western world.^1^ To date, no medical therapies are available, making treatment options limited to surgical or endovascular replacement with a biological or mechanical prosthetic valve. However, biological valves have limited durability due to gradual structural deterioration eventually leading to graft failure, and mechanical valves necessitate life-long treatment with potent anticoagulants.^2^ The ideal heart valve replacement is considered to be an off-the-shelf valve that does not exert an immune response and has the capacity to grow and be repopulated by living cells. Therefore, tissue-engineered heart valves, both decellularized and *in vitro*-produced scaffolds, have been developed and extensively studied in animal models,^3–9^ and decellularized aortic valve allografts have even reached clinical trials.^10,11^ Although initial studies have been favorable, tissue-engineered heart valves suffer from structural deterioration similar to conventional allografts and may even be associated with a higher reoperation rate.^12^ What causes degeneration of tissue-engineered valves is unknown.^12,13^

Despite the widely recognized importance of the extracellular matrix (ECM), and the significant interest in heart valve tissue engineering, the important basement membrane (BM) of heart valves remains poorly characterized. The BM plays key roles in cell adhesion, migration, function, and differentiation.^14^ Laminins, consisting of an α, β, and γ chain, are the first BM components to be expressed in mammalian tissues.^15^ Their importance in cell function and tissue formation is illustrated by the fact that several laminin chain isoforms are vital for normal embryonic development.^14^ However, the laminin isoforms and their expression pattern in heart valves have not been characterized, and the role of laminins in tissue engineering remains largely unknown.

Decellularized scaffolds provide an opportunity to investigate the effects of individual ECM components in a regenerative context relevant for tissue engineering. They have previously been used to study how heparan sulfate proteoglycans affect the differentiation of endoderm-derived stem cells on a decellularized lung scaffold.^16^ However, it is well known that different tissues require different methods for decellularization^17–20^ as the process inevitably has detrimental effects on the ECM.^21^ Therefore, optimization of a decellularization protocol for the specific tissue in question to achieve effective cell removal while limiting detergent exposure is critical.

The aim of the present study was two-fold. First, to characterize the laminin isoforms present in human and rat aortic valves, and their distribution patterns. Second, to develop a decellularized rat aortic valve scaffold that preserves laminin and other BM components for future studies on how the BM influences cell behavior in heart valve regeneration.

## Materials and Methods

### Human tissues

Whole human hearts were obtained from Legacy Donor Services Foundation, Florida. Legacy Donor Services Foundation receives donor referrals from hospitals and medical examiners serving the state of Florida. Written informed consent was obtained from the donor through registration as a tissue and organ donor with Donate Life America, Donate Life Florida, or registering to be a donor when renewing or obtaining a driver's license. Alternatively, written informed consent was obtained from the donor's next-of-kin by a Donor Management Coordinator from Legacy Donor Services Foundation. Hearts intended for biomedical research were offered to Nova Southeastern University by the Legacy Donor Services Foundation. Inclusion criteria were donor age 18–60 years and no history of valvular heart disease. Institutional Review Board approval was obtained from the Regional Ethics Review Committee in Stockholm, Sweden (Protocol Number: 2017/2268-31). Hearts (*n* = 3) were processed within 48 h from the time of death. The aortic valve was inspected for gross morphologic abnormalities, the cusps were excised at their origin and snap frozen in optimal cutting temperature (OCT) compound.

### Animals

Rat aortic roots were procured from female Sprague–Dawley rats 8–12 weeks of age (Charles River). All animals were cared for in accordance with the rules and regulations of Karolinska University Hospital and Karolinska Institutet. Institutional Review Board approvals for animal experiments were obtained from the Regional Committee on the Ethics of Animal Experiments, Linköping, Sweden (Protocol Numbers: ID 6-17 and S190-12).

### Rat aortic valve procurement

Rats were anesthetized through isoflurane inhalation and then immediately euthanized by inhalation of CO_2_ or decapitation. The thoracic cavity was accessed by dividing the ribs and sternoclavicular joints and excising the sternum. All intrathoracic organs were removed en bloc. The aortic valve was dissected from the heart under a microscope as a U-shaped aortic conduit as described by others.^22,23^ Briefly, a myocardial cuff of ∼1–2 mm and part of the anterior mitral valve leaflet were preserved, whereas the brachiocephalic artery, left common carotid artery, and left subclavian artery were ligated with 6-0 sutures (Ethicon) at their origin. The descending aorta was divided at the level of the aortic valve. For normal controls, the ascending aorta was divided at the origin of the brachiocephalic artery and the aortic root was then embedded in OCT and snap frozen.

### Decellularization

A perfusion decellularization method based on earlier protocols^5,17^ was used. In brief, the aortic conduit was cannulated with a 1.1 mm intravenous catheter (B. Braun Medical), which was connected to a tubing pump (Ismatec) through a custom-built stainless steel bottle cap to enable recirculation of reagents in a 250-mL bottle (Supplementary Fig. S1A). Aortic conduits were perfused with 1% sodium dodecyl sulfate (SDS; Merck Sigma-Aldrich) in deionized water for 3 h at a flow rate of 2 mL/min, followed by 15 min with deionized water, 30 min with 1% Triton X-100 (Merck Sigma-Aldrich) in deionized water and 12–24 h with phosphate-buffered saline (PBS), changed twice. The aorta was divided at the origin of the brachiocephalic artery to facilitate enzymatic treatment. Removal of residual nucleic acids was achieved by processing aortic roots with 90 U benzonase endonuclease (E1014; Merck Sigma-Aldrich) on a see-saw rocker (Cole-Parmer) overnight at room temperature (RT). The remaining aortic roots were processed on the see-saw rocker overnight in PBS and then snap frozen in OCT or used for DNA quantification.

### Histology and immunohistochemistry

Human valve cusps (*n* = 3), and normal (*n* = 3) and decellularized (*n* = 3) rat aortic roots were sectioned to a thickness of 5 and 8 μm, respectively, using a cryostat (Leica). Slides were fixed in 4% formaldehyde and stained with Hematoxylin and Eosin (H&E) using standard methods. For immunohistochemistry, slides were fixed in 4% formaldehyde (or 4% paraformaldehyde), methanol, or acetone. When appropriate, samples were boiled in citrate buffer pH 6 or Diva decloaker (Biocare Medical) for 4.5 min and cooled at RT. Slides were then blocked with 5% donkey serum (D9663; Merck Sigma-Aldrich), 5% goat serum (S-1000; Vector Laboratories, PCN5000; Thermo Fisher), 5% rabbit serum (X0902; Dako), or 1% bovine serum albumin fraction V (K41-001; PAA Laboratories) for 30 min, followed by primary antibody incubation overnight in RT. Antibodies against collagen I (Ab34710), elastin (clone BA-4; Abcam), laminin α1 (AF4187; R&D Systems), α2 (clone CL3450), α3 (clone CL3112), α4 (clone CL3183), β2 (clone CL2976), β3 (clone CL3353), γ1 (clone CL3199), γ2 (clone CL2980; Atlas Antibodies), α5 (clone 4C7), and β1 (clone 4E10; Millipore) were used on human samples. Rat tissue sections were incubated with antibodies against laminin α4 (clone CL3183), α5 (C13068; Assay Biotechnology, Supplementary Fig. S2), γ1 (clone 2E8; Millipore), perlecan (clone 11B4^24^), heparan sulfate (clone F58-10E4; Amsbio), collagen IV (Ab6586), and fibronectin (Ab23750; Abcam). A detailed overview of immunohistochemistry protocols is provided in Supplementary Table S1. The slides were then incubated with one of the following Alexa Fluor-conjugated secondary antibodies for 2 h in RT: donkey anti-rabbit immunoglobulin G (IgG, A-10040; Thermo Fisher), goat anti-mouse IgG (A-11004; Thermo Fisher), goat anti-mouse immunoglobulin M (A-21042; Thermo Fisher), goat anti-rabbit IgG (A-11008; Thermo Fisher), rabbit anti-mouse IgG (A-11059; Thermo Fisher), or donkey anti-goat IgG (ab175704; Abcam). Nuclei were counterstained with 4,6-diamidino-2-phenylindole (DAPI). Rat aortic valves were visualized with a fluorescence microscope (Olympus; Leica) with an attached camera (Olympus; QImaging). Human tissue sections were visualized with a laser scanning confocal microscope (Zeiss; Leica).

### DNA quantification

DNA content per decellularized aortic valve (*n* = 3) was compared with normal controls (*n* = 6). To provide an estimation of residual cellular contents other than nucleic acids, aortic valves treated with SDS for 3 h, but not processed with benzonase endonuclease, were included (*n* = 3). Aortic roots treated with SDS for 12 h (*n* = 3) were also included to quantify difference in residual cellular contents and nucleic acids between the original protocol^17^ and our modified protocol. The ascending aorta was opened longitudinally along one of the commissures and the aortic valve leaflets were excised in their entirety under a microscope. DNA was extracted using the DNeasy Blood and Tissue Kit (Qiagen) according to the manufacturer's instructions. Purified DNA was eluted twice in 25 μL buffer and quantified with NanoDrop (Implen).

### Statistics

One-way analysis of variance with Bonferroni corrections for multiple comparisons was used to identify differences between groups. *p* < 0.05 was considered statistically significant.

## Results

### Healthy human aortic valves express laminin α4, α5, β1, β2, and γ1 chains

**FIG. 1.**
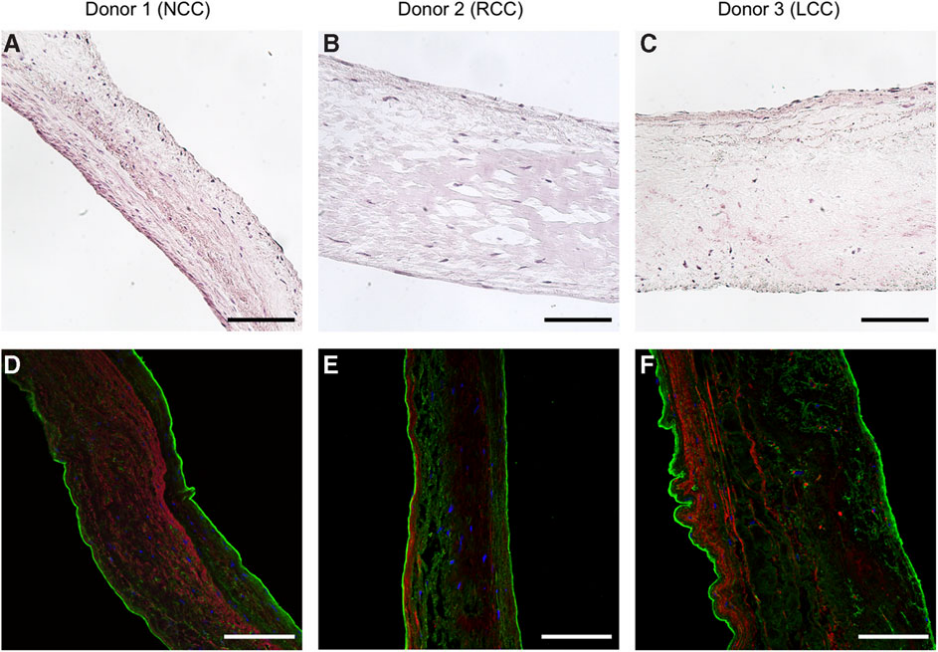
Histology of donor aortic valve cusps. **(A**–**C)** H&E. **(D**–**F)** Collagen I (green) and elastin (red), nuclei counterstained with DAPI (blue). All three cusps had an intact three-layered structure and an overall normal histology. Elastin identifies the ventricularis **(D**–**F)**, as well as the interstitial vessels **(D)**. A representative image of four **(A**–**C)** or one **(D**–**F)** tissue sections is shown. All images were captured with 20 × magnification. Scale bar represents 100 μm. DAPI, 4,6-diamidino-2-phenylindole; NCC, noncoronary cusp; RCC, right coronary cusp; LCC, left coronary cusp; H&E, Hematoxylin and Eosin.

Aortic valves were from male donors 50–60 years of age. Upon gross morphologic assessment, one of the hearts was hypertrophic and had significant atherosclerosis in the main coronary arteries (Table 1). All three aortic valves were tricuspid and macroscopically normal. A single aortic valve cusp was used from each donor, that is, one right coronary cusp, one left coronary cusp, and one noncoronary cusp. H&E staining and double staining for elastin and collagen I confirmed the absence of plaques and an overall normal histology with an intact three-layered structure (Fig. 1). The noncoronary cusp had a markedly vascularized interstitium, the left coronary- and right noncoronary cusp had limited or no vascularization.

**Table 1.** Donor Demographics and Heart Morphology

Donor	Sex (M/F)	Age (years)	Significant CAD	Significant ventricular hypertrophy	Cusp assessed
1	M	50	Yes	Yes	NCC
2	M	55	No	No	RCC
3	M	50	No	No	LCC

M, male; F, female; CAD, coronary artery disease; NCC, noncoronary cusp; RCC, right coronary cusp; LCC, left coronary cusp.

**FIG. 2.**
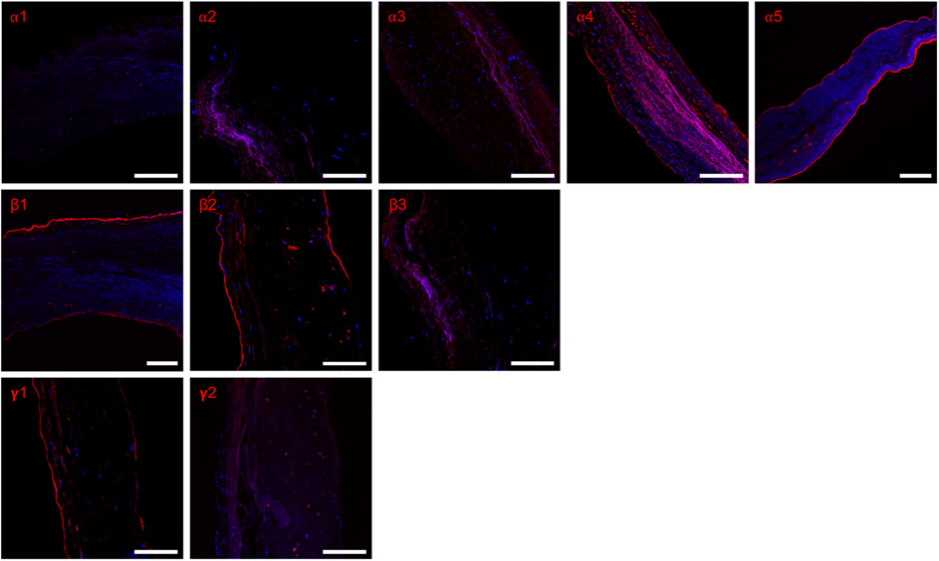
Laminin chain expression in human aortic valves. The endothelial basement membrane covering the valve cusps were positive for laminin α4, α5, β1, β2, and γ1, as well as the endothelial lining of interstitial vessels and minor patches in the interstitium. Images of α1, α2, α3, β3, and γ2 show absence of immunoreactivity. A representative image of ≥3 tissue sections from each of the three donor valve cusps is shown. All images shown at 20 × magnification. Scale bar represents 100 μm.

The endothelial BM of the human aortic valve cusps stained positive for laminin α4, α5, β1, β2, and γ1 chains (Fig. 2). All chains were uniformly present in all three cusps, forming a continuous monolayer. The same chains were also expressed in the BM of the interstitial vessels observed in two of the cusps. The interstitium itself was mostly negative for the laminin chains, except in minor regions. Laminin α1, α2, α3, β3, and γ2 could not be detected in any of the valve cusps. This suggests that the human aortic valve endothelial BM consists of LN-411, LN-421, LN-511, and LN-521.

**FIG. 3.**
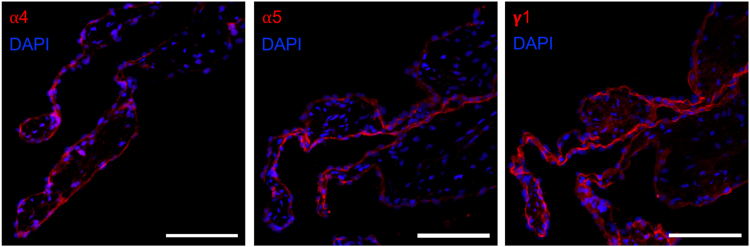
Expression of laminin α4, α5, and γ1 in rat aortic valves. The endothelial basement membrane stained positive for all three laminin chains. Minimal immunoreactivity was seen in the interstitium. A representative image of ≥4 tissue sections from three animals per group is shown. All images captured with 20 × magnification. Scale bar represents 100 μm.

To assess the relevance and utility of the rat model, three rat aortic roots were stained for laminin α4, α5, and γ1. Rat aortic valve cusps also stained positive for α4, α5, and γ1, indicating the presence of the same laminin isoforms as in human valves (Fig. 3). The distribution pattern was uniform for all three laminin chains, which formed a convergent monolayer, continuous with the endocardium and aortic endothelium.

### Decellularization with SDS and Triton X-100 preserves BM components

**FIG. 4.**
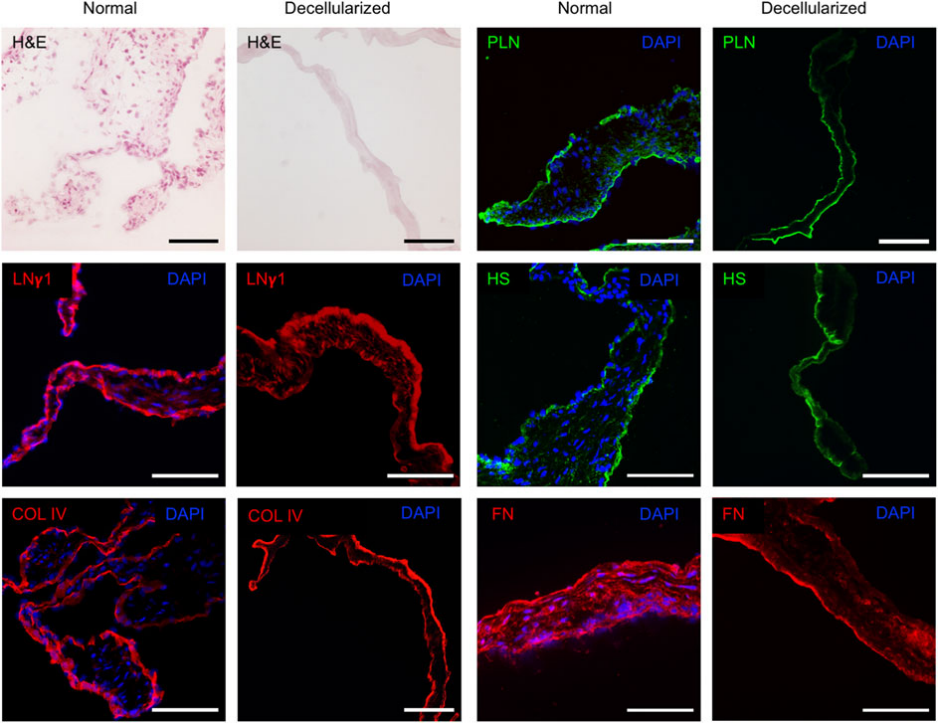
Histology of normal and decellularized rat aortic valves. H&E images and DAPI staining of cell nuclei reveal absence of intact nuclei after decellularization. Laminin γ1, collagen IV, perlecan, heparan sulfate, and fibronectin was visualized in both normal controls and decellularized valves, with a largely preserved distribution pattern after decellularization. A representative image of ≥3 tissue sections from three animals per group is shown. All images were captured at 20 × magnification. Scale bar represents 100 μm. LNγ1, laminin γ1; COL IV, collagen IV; PLN, perlecan; HS, heparan sulfate; FN, fibronectin.

To establish a small animal model for studies of cell–laminin interactions, we sought to assess the effectiveness and effects on the BM of a decellularization protocol for rat aortic valves adapted from earlier studies.^5,17^ Aortic roots were exposed to 1% SDS for 1, 3, 6, or 12 h (*n* = 1 for each time point). After 3 h, the myocardial rim remained incompletely decellularized macroscopically, whereas the entire specimen was completely translucent after 12 h (Supplementary Fig. S1B–D). No clear difference could be observed in terms of DAPI-positive intact nuclei or residual DNA in the valve cusps at the different time points, (Supplementary Fig. S3) and intact nuclei could still be seen in the aortic annulus even after 12 h. To ensure adequate SDS treatment while avoiding prolonged exposure, 3 h was chosen as an intermediate incubation time for further assessment. As residual DNA has been associated with an adverse immune response,^25^ further processing with benzonase endonuclease was performed,^16,18^ resulting in an almost complete removal of nucleic acids in the valve (Fig. 4). Occasionally, isolated intact cell nuclei could be observed in the valve cusps (Supplementary Fig. S3).

To confirm the integrity of the BM, tissue sections of decellularized rat aortic valves (*n* = 3) and normal controls (*n* = 3) were stained for laminin γ1, common to all laminin isoforms found in the aortic valve, as well as perlecan, heparan sulfate, and collagen IV (Fig. 4). All of the analyzed BM components, as well as fibronectin, could be visualized after decellularization, with a largely intact distribution pattern.

### Limited detergent exposure combined with benzonase endonuclease results in minimal residual DNA

**FIG. 5.**
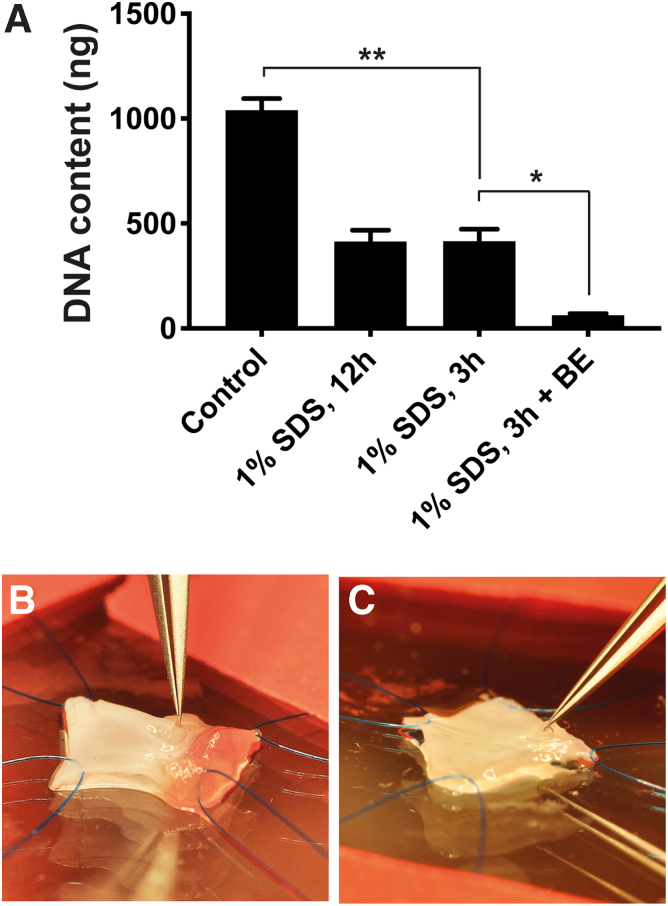
Quantification of DNA in rat aortic valves before and after decellularization. **(A)** DNA content in normal controls (*n* = 6) and decellularized valves treated with SDS for 3 h (*n* = 3), 12 h (*n* = 3), or 3 h with additional treatment with BE (*n* = 3). **(B**, **C)** Dissection technique for excision of intact valve cusps in normal control **(B)** and decellularized aortic root **(C)**. *p < 0.05. **p < 0.0001. SDS, sodium dodecyl sulfate; BE, benzonase endonuclease.

Due to their extremely small mass, we were unable to obtain reliable weight measurements from the valve cusps. However, by using fine dissection tools, the three valve cusps could be excised from the aortic root in a highly reproducible fashion, enabling assessment of DNA content per valve, that is, all three cusps excised from the same specimen. Normal control aortic valves (*n* = 6) had a mean DNA content of 1.04 μg (Fig. 5; Supplementary Table S2). This was reduced to 413.8 ng in aortic valves after 3 h of SDS perfusion (39.8% of normal controls, *p* < 0.001). The DNA content was similarly reduced to 414.4 ng after 12 h of SDS perfusion (39.8% of normal controls, *p* < 0.001). Additional processing with benzonase endonuclease resulted in a mean aortic valve DNA content of 63.4 ng (6.1% of normal controls), a significant decrease compared with 3 h SDS exposure without enzymatic treatment (*p* = 0.0137).

## Discussion

The present study describes, for the first time, the specific laminin chains present in healthy human aortic valves. We show that the main laminin isoforms expressed in the endothelial BM of heart valves are LN-411, LN-421, LN-511, and LN-521, and that the isoforms are conserved across mammalian species. The same laminin isoforms were also expressed in small interstitial vessels that may exist in valve cusps.^26,27^ Interestingly, small traces of laminin that appeared to be located in the interstitium rather than in the endothelial BM were observed. These findings are consistent with previous reports on laminin isoforms expressed in vascular endothelial BMs.^28–33^ However, cardiac valvular cells and their ECM are unique in structure and function,^34–36^ and our study provides critical data on the BM structure and microenvironment of valvular cells necessary for recreating the native BM in tissue-engineered heart valves.

Laminins are key components of vascular BMs and have important, isoform-specific functions.^37^ Laminin α4 is ubiquitous in the BM of blood vessels and most endothelial BMs also express α5.^29–31^ However, their distribution pattern and relative expression levels vary considerably between different vessel types, which affects the properties of the BM. For example, the expression patterns of α4 and α5 are known to determine the tightness of endothelial junctions and thus the permeability of the endothelium in the context of lymphocyte extravasation, with α4 isoforms being permissive and α5 isoforms being restrictive.^38–41^ Furthermore, evidence suggests that α4 has a role in angiogenesis as well as the regulation of endothelial cell survival,^42,43^ indicating a role in endothelialization of tissue-engineered valves and vascular grafts. Indeed, laminin coating, although of an undefined isoform, has been shown to promote endothelialization while decreasing intimal hyperplasia in aortic grafts *in vivo*.^6^ On the other hand, the α5-containing LN-511 has been shown to be important in the shear stress response of resistance arteries^44^ and may be of similar importance in heart valves during the cardiac cycle. The β-chain is also significant in endothelial BMs as an isoform switch from LN-421 to LN-411 may lead to decreased adhesion and increased endothelial-to-mesenchymal transition.^33^ Furthermore, laminins may have additional regulatory effects on valvular interstitial cells.^45,46^

In conclusion, laminins play key roles in the cardiovascular system, and the presence or lack of specific laminin isoforms in tissue-engineered heart valves may thus have a profound impact on their viability. However, the role of laminins in tissue engineering needs to be defined to assess their true relevance in this context, and further studies on small animal scaffolds are an appropriate next step. While decellularized rat aortic valves have been described by others,^5,23,47–49^ we present a protocol with reduced detergent exposure, which results in both effective cell removal and preservation of laminin and other key BM components. Scaffolds produced through limited detergent exposure, and thus with a better preserved BM,^20,21,50^ will be a useful tool in future mechanistic studies.

The number of donated heart valves in our study was limited due to the sparse availability of healthy human heart valves. We did not obtain clinical data, such as cause of death, comorbidities, and clinical risk factors, which could have added insights into whether valves were at risk of calcific degeneration. However, all valves had a normal gross morphology and histology. Furthermore, the laminin expression in all human aortic valves was fully consistent between specimens, and data are supported by current literature on laminins in the cardiovascular system.^28–33,37^

To the best of our knowledge, this is the first report to describe the laminin chains expressed in aortic valves and their localization. Given the importance of laminins for a number of cellular functions, this provides crucial data for future attempts to recreate the aortic valve through tissue engineering. The presence or absence of laminins, and other ECM components such as perlecan,^51–54^ heparan sulfate,^16^ and fibronectin^4,55^ is bound to affect the viability of tissue-engineered heart valve grafts, but other factors that influence graft viability also need to be considered. Aside from a lack of appropriate ECM components, structural deterioration of tissue-engineered heart valve grafts may be due to (i) immunogenicity,^56^ (ii) thrombogenicity,^57^ or (iii) incomplete or inappropriate valvular endothelial or interstitial cell repopulation.^3,4,8^ The relative importance of laminins and other ECM components, as well as other factors that may affect the long-term viability of a tissue-engineered heart valve, will need to be determined in future studies.

## Conclusion

We have shown that the aortic valve BM contains LN-411, LN-421, LN-511, and LN-521. We also describe a modified decellularized rat scaffold with a largely preserved BM for future mechanistic studies. Further studies are required to quantify the laminin isoforms and determine their role in heart valve tissue engineering. Additional studies are also necessary to assess the effect of detergents used during decellularization on the functional properties of ECM components in heart valve regeneration.

## Supplementary Material

Supplemental data

Supplemental data

Supplemental data

Supplemental data

Supplemental data

## Abbreviations Used

BE: benzonase endonuclease
BM: basement membrane
CAD: coronary artery disease
COL IV: collagen IV
DAPI: 4,6-diamidino-2-phenylindole
ECM: extracellular matrix
FN: fibronectin
H&E: hematoxylin and eosin
HS: heparan sulfate
IgG: immunoglobulin G
LCC: left coronary cusp
LNγ1: laminin γ1
NCC: noncoronary cusp
PBS: phosphate-buffered saline
PLN: perlecan
RCC: right coronary cusp
RT: room temperature
SDS: sodium dodecyl sulfate

## References

[1] Iung B, Baron G, Butchart EG, et al. A prospective survey of patients with valvular heart disease in Europe: the Euro Heart Survey on valvular heart disease. Eur Heart J. 2003;24:1231–1243 12831818 10.1016/s0195-668x(03)00201-x

[2] Nishimura RA, Otto CM, Bonow RO, et al. 2014 AHA/ACC guideline for the management of patients with valvular heart disease: a report of the American college of cardiology/American heart association task force on practice guidelines. J Am Coll Cardiol. 2014;63:e57–e185 24603191 10.1016/j.jacc.2014.02.536

[3] Tudorache I, Theodoridis K, Baraki H, et al. Decellularized aortic allografts versus pulmonary autografts for aortic valve replacement in the growing sheep model: haemodynamic and morphological results at 20 months after implantation. Eur J Cardiothorac Surg. 2016;49:1228–1238 26503725 10.1093/ejcts/ezv362

[4] Flameng W, De Visscher G, Mesure L, et al. Coating with fibronectin and stromal cell-derived factor-1-alpha of decellularized homografts used for right ventricular outflow tract reconstruction eliminates immune response-related degeneration. J Thorac Cardiovasc Surg. 2014;147:1398–1404.e2. 23896322 10.1016/j.jtcvs.2013.06.022

[5] Grauss RW, Hazekamp MG, Van Vliet S, et al. Decellularization of rat aortic valve allografts reduces leaflet destruction and extracellular matrix remodeling. J Thorac Cardiovasc Surg. 2003;126:2003–2010 14688719 10.1016/s0022-5223(03)00956-5

[6] Toshmatova M, Nakanishi S, Sugimura Y, et al. Influence of laminin coating on the autologous in vivo recellularization of decellularized vascular protheses. Materials (Basel). 2019;12:3351 10.3390/ma12203351 PMC6829566 31618810

[7] Iop L, Bonetti A, Naso F, et al. Decellularized allogeneic heart valves demonstrate self-regeneration potential after a long-term preclinical evaluation. PLoS One. 2014;9:e99593 24940754 10.1371/journal.pone.0099593 PMC4062459

[8] Syedain Z, Reimer J, Schmidt J, et al. 6-Month aortic valve implantation of an off-the-shelf tissue-engineered valve in sheep. Biomaterials. 2015;73:175–184 26409002 10.1016/j.biomaterials.2015.09.016 PMC5520964

[9] Weber B, Dijkman PE, Scherman J, et al. Off-the-shelf human decellularized tissue-engineered heart valves in a non-human primate model. Biomaterials. 2013;34:7269–7280 23810254 10.1016/j.biomaterials.2013.04.059

[10] Tudorache I, Horke A, Cebotari S, et al. Decellularized aortic homografts for aortic valve and aorta ascendens replacement. Eur J Cardiothorac Surg. 2016;50:89–97 26896320 10.1093/ejcts/ezw013 PMC4913875

[11] Da Costa FDA, Costa ACBA, Prestes R, et al. The early and midterm function of decellularized aortic valve allografts. Ann Thorac Surg. 2010;90:1854.–1860 21095325 10.1016/j.athoracsur.2010.08.022

[12] Helder MRKK, Kouchoukos NT, Zehr K, et al. Late durability of decellularized allografts for aortic valve replacement: a word of caution. J Thorac Cardiovasc Surg. 2016;152:1197–1199 27131847 10.1016/j.jtcvs.2016.03.050

[13] Blum KM, Drews JD, Breuer CK Tissue-engineered heart valves: a call for mechanistic studies. Tissue Eng Part B Rev. 2018;24:240–253 29327671 10.1089/ten.teb.2017.0425 PMC5994154

[14] Domogatskaya A, Rodin S, Tryggvason K Functional diversity of laminins. Annu Rev Cell Dev Biol. 2012;28:523–553 23057746 10.1146/annurev-cellbio-101011-155750

[15] Leivo I, Vaheri A, Timpl R, et al. Appearance and distribution of collagens and laminin in the early mouse embryo. Dev Biol. 1980;76:100–114 6991310 10.1016/0012-1606(80)90365-6

[16] Shojaie S, Ermini L, Ackerley C, et al. Acellular lung scaffolds direct differentiation of endoderm to functional airway epithelial cells: requirement of matrix-bound HS proteoglycans. Stem Cell Rep. 2015;4:419–430 10.1016/j.stemcr.2015.01.004 PMC4375883 25660407

[17] Ott HC, Matthiesen TS, Goh S-K, et al. Perfusion-decellularized matrix: using nature's platform to engineer a bioartificial heart. Nat Med. 2008;14:213–221 18193059 10.1038/nm1684

[18] Petersen TH, Calle EA, Zhao L, et al. Tissue-engineered lungs for in vivo implantation. Science 2010;329:538–541 20576850 10.1126/science.1189345 PMC3640463

[19] Baptista PM, Siddiqui MM, Lozier G, et al. The use of whole organ decellularization for the generation of a vascularized liver organoid. Hepatology. 2011;53:604–617 21274881 10.1002/hep.24067

[20] Crapo PM, Gilbert TW, Badylak SF An overview of tissue and whole organ decellularization processes. Biomaterials. 2011;32:3233–3243 21296410 10.1016/j.biomaterials.2011.01.057 PMC3084613

[21] Faulk DM, Carruthers CA, Warner HJ, et al. The effect of detergents on the basement membrane complex of a biologic scaffold material. Acta Biomater. 2014;10:183–193 24055455 10.1016/j.actbio.2013.09.006 PMC3857635

[22] Oei FB, Welters MJ, Bonthuis F, et al. A size-matching heterotopic aortic valve implantation model in the rat. J Surg Res. 1999;87:239–244 10600355 10.1006/jsre.1999.5763

[23] Assmann A, Akhyari P, Delfs C, et al. Development of a growing rat model for the in vivo assessment of engineered aortic conduits. J Surg Res. 2012;176:367–375 22172135 10.1016/j.jss.2011.10.009

[24] Jiang X, Multhaupt H, Chan E, et al. Essential contribution of tumor-derived perlecan to epidermal tumor growth and angiogenesis. J Histochem Cytochem. 2004;52:1575–1590 15557212 10.1369/jhc.4A6353.2004

[25] Keane TJ, Londono R, Turner NJ, et al. Consequences of ineffective decellularization of biologic scaffolds on the host response. Biomaterials. 2012;33:1771–1781 22137126 10.1016/j.biomaterials.2011.10.054

[26] Weind KL, Ellis CG, Boughner DR Aortic valve cusp vessel density: relationship with tissue thickness. J Thorac Cardiovasc Surg. 2002;123:333–340 11828294 10.1067/mtc.2002.119696

[27] Kholová I, Dragneva G, Čermáková P, et al Lymphatic vasculature is increased in heart valves, ischaemic and inflamed hearts and in cholesterol-rich and calcified atherosclerotic lesions. Eur J Clin Invest. 2011;41:487–497 21128936 10.1111/j.1365-2362.2010.02431.x

[28] Iivanainen A, Kortesmaa J, Sahlberg C, et al. Primary structure, developmental expression, and immunolocalization of the murine laminin α4 chain. J Biol Chem. 1997;272:27862–27868 9346933 10.1074/jbc.272.44.27862

[29] Sorokin L, Girg W, Göpfert T, et al. Expression of novel 400-kDa laminin chains by mouse and bovine endothelial cells. Eur J Biochem. 1994;223:603–610 8055931 10.1111/j.1432-1033.1994.tb19031.x

[30] Frieser M, Nöckel H, Pausch F, et al. Cloning of the mouse laminin α4 cDNA. Expression in a subset of endothelium. Eur J Biochem. 1997;246:727–735 9219532 10.1111/j.1432-1033.1997.t01-1-00727.x

[31] Sorokin LM, Pausch F, Frieser M, et al. Developmental regulation of the laminin chain suggests a role in epithelial and endothelial cell maturation. Dev Biol. 1997;189:285–300 9299121 10.1006/dbio.1997.8668

[32] Sasaki T, Mann K, Miner JH, et al. Domain IV of mouse laminin β1 and β2 chains: structure, glycosaminoglycan modification and immunochemical analysis of tissue contents. Eur J Biochem. 2002;269:431–442 11856301 10.1046/j.0014-2956.2001.02663.x

[33] Wagner JUG, Chavakis E, Rogg EM, et al. Switch in laminin β2 to laminin β1 isoforms during aging controls endothelial cell functions-brief report. Arterioscler Thromb Vasc Biol. 2018;38:1170–1177 29599141 10.1161/ATVBAHA.117.310685

[34] Hinton RB, Lincoln J, Deutsch GH, et al. Extracellular matrix remodeling and organization in developing and diseased aortic valves. Circ Res. 2006;98:1431–1438 16645142 10.1161/01.RES.0000224114.65109.4e

[35] Butcher JT, Tressel S, Johnson T, et al. Transcriptional profiles of valvular and vascular endothelial cells reveal phenotypic differences: influence of shear stress. Arterioscler Thromb Vasc Biol. 2006;26:69–77 16293796 10.1161/01.ATV.0000196624.70507.0d

[36] Butcher JT, Penrod AM, García AJ, et al. Unique morphology and focal adhesion development of valvular endothelial cells in static and fluid flow environments. Arterioscler Thromb Vasc Biol. 2004;24:1429–1434 15117733 10.1161/01.ATV.0000130462.50769.5a

[37] Di Russo J, Hannocks MJ, Luik AL, et al. Vascular laminins in physiology and pathology. Matrix Biol. 2017;57–58:140–148 10.1016/j.matbio.2016.06.008 27378388

[38] Wu C, Ivars F, Anderson P, et al. Endothelial basement membrane laminin α5 selectively inhibits T lymphocyte extravasation into the brain. Nat Med. 2009;15:519–527 19396173 10.1038/nm.1957

[39] Song J, Zhang X, Buscher K, et al. Endothelial basement membrane laminin 511 contributes to endothelial junctional tightness and thereby inhibits leukocyte transmigration. Cell Rep. 2017;18:1256–1269 28147279 10.1016/j.celrep.2016.12.092

[40] Sixt M, Hallmann R, Wendler O, et al. Cell adhesion and migration properties of β2-integrin negative polymorphonuclear granulocytes on defined extracellular matrix molecules: relevance for leukocyte extravasation. J Biol Chem. 2001;276:18878–18887 11278780 10.1074/jbc.M010898200

[41] Thyboll J, Kortesmaa J, Cao R, et al. Deletion of the laminin 4 chain leads to impaired microvessel maturation. Mol Cell Biol. 2002;22:1194–1202 11809810 10.1128/MCB.22.4.1194-1202.2002 PMC134646

[42] Gonzalez AM, Gonzales M, Herron GS, et al. Complex interactions between the laminin α4 subunit and integrins regulate endothelial cell behavior in vitro and angiogenesis in vivo. Proc Natl Acad Sci U S A. 2002;99:16075–16080 12454288 10.1073/pnas.252649399 PMC138567

[43] Knöll R, Postel R, Wang J, et al. Laminin-α4 and integrin-linked kinase mutations cause human cardiomyopathy via simultaneous defects in cardiomyocytes and endothelial cells. Circulation. 2007;116:515 17646580 10.1161/CIRCULATIONAHA.107.689984

[44] Di Russo J, Luik A, Yousif L, et al. Endothelial basement membrane laminin 511 is essential for shear stress response. EMBO J. 2017;36:1464 28507085 10.15252/embj.201797000 PMC5430226

[45] Gu X, Masters KS Regulation of valvular interstitial cell calcification by adhesive peptide sequences. J Biomed Mater Res Part A. 2010;93:1620–1630 10.1002/jbm.a.32660 PMC2860685 20073077

[46] Monzack EL, Gu X, Masters KS Efficacy of simvastatin treatment of valvular interstitial cells varies with the extracellular environment. Arterioscler Thromb Vasc Biol. 2009;29:246–253 19023089 10.1161/ATVBAHA.108.179218 PMC2701301

[47] Assmann A, Struss M, Schiffer F, et al. Improvement of the in vivo cellular repopulation of decellularized cardiovascular tissues by a detergent-free, non-proteolytic, actin-disassembling regimen. J Tissue Eng Regen Med. 2017;11:3530–3543 28078820 10.1002/term.2271

[48] Kallenbach K, Sorrentino S, Mertsching H, et al. A novel small-animal model for accelerated investigation of tissue-engineered aortic valve conduits. Tissue Eng Part C Methods. 2010;16:41–50 19351241 10.1089/ten.TEC.2008.0595

[49] Meyer SR, Chiu B, Churchill TA, et al. Comparison of aortic valve allograft decellularization techniques in the rat. J Biomed Mater Res Part A. 2006;79:254–262 10.1002/jbm.a.30777 16817222

[50] Akhyari P, Aubin H, Gwanmesia P, et al. The quest for an optimized protocol for whole-heart decellularization: a comparison of three popular and a novel decellularization technique and their diverse effects on crucial extracellular matrix qualities. Tissue Eng Part C Methods. 2011;17:915–926 21548726 10.1089/ten.TEC.2011.0210

[51] Nugent MA, Nugent HM, Iozzo RV, et al. Perlecan is required to inhibit thrombosis after deep vascular injury and contributes to endothelial cell-mediated inhibition of intimal hyperplasia. Proc Natl Acad Sci U S A. 2000;97:6722–6727 10841569 10.1073/pnas.97.12.6722 PMC18716

[52] Tran PK, Tran-Lundmark K, Soininen R, et al. Increased intimal hyperplasia and smooth muscle cell proliferation in transgenic mice with heparan sulfate-deficient perlecan. Circ Res. 2004;94:550–558 14739157 10.1161/01.RES.0000117772.86853.34

[53] Gotha L, Lim SY, Osherov AB, et al. Heparan sulfate side chains have a critical role in the inhibitory effects of perlecan on vascular smooth muscle cell response to arterial injury. Am J Physiol Heart Circ Physiol 2014;307:H337–H345 24858854 10.1152/ajpheart.00654.2013

[54] Costell M, Carmona R, Gustafsson E, et al. Hyperplastic conotruncal endocardial cushions and transposition of great arteries in perlecan-null mice. Circ Res. 2002;91:158–164 12142349 10.1161/01.res.0000026056.81424.da

[55] Assmann A, Delfs C, Munakata H, et al. Acceleration of autologous invivo recellularization of decellularized aortic conduits by fibronectin surface coating. Biomaterials. 2013;34:6015–6026 23683757 10.1016/j.biomaterials.2013.04.037

[56] Sarikouch S, Theodoridis K, Hilfiker A, et al. Early insight into in vivo recellularization of cell-free allogenic heart valves. Ann Thorac Surg. 2019;108:581–589 30928547 10.1016/j.athoracsur.2019.02.058

[57] Baraki H, Tudorache I, Braun M, et al. Orthotopic replacement of the aortic valve with decellularized allograft in a sheep model. Biomaterials. 2009;30:6240–6246 19703713 10.1016/j.biomaterials.2009.07.068

