# Supplementary material for: Characterization of Laminins in Healthy Human Aortic Valves and a Modified Decellularized Rat Scaffold
Source: Biores Open Access. 2020 Dec 7;9(1):269–78. doi: 10.1089/biores.2020.0018 (PMC7757704; doi:10.1089/biores.2020.0018)
Supplement: Supplemental data [file Supp_Fig1.docx]

**
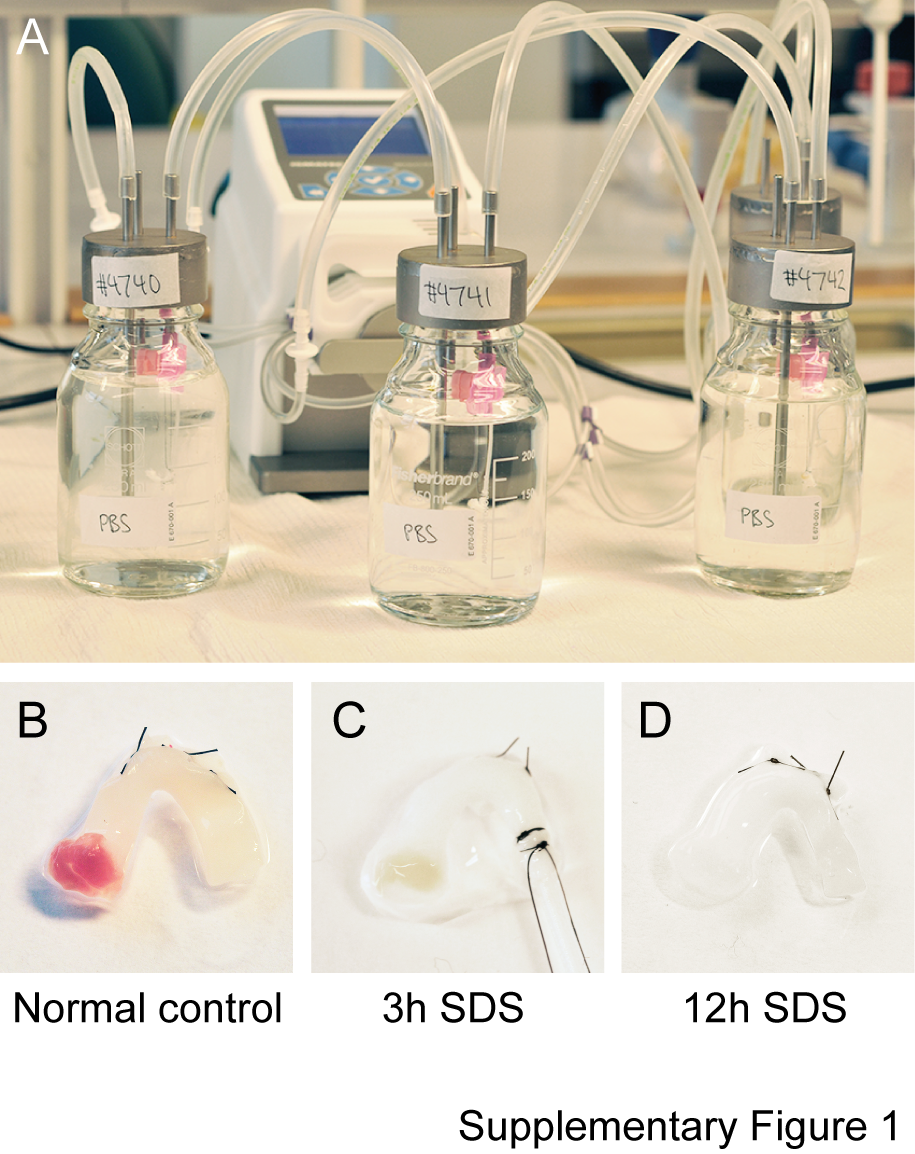
**

**Fig S1. Decellularization setup and macroscopic images of rat aortic roots.**

(A) Setup for perfusion decellularization using a custom-built stainless-steel bottle cap and a tubing pump (Ismatec). (B) Normal untreated control. (C) Aortic root processed with 1% SDS for 3 h. (D) Aortic root processed with 1% SDS for 12 h. SDS = Sodium dodecyl sulfate.
