# Supplementary material for: Characterization of Laminins in Healthy Human Aortic Valves and a Modified Decellularized Rat Scaffold
Source: Biores Open Access. 2020 Dec 7;9(1):269–78. doi: 10.1089/biores.2020.0018 (PMC7757704; doi:10.1089/biores.2020.0018)
Supplement: Supplemental data [file Supp_Fig2.docx]

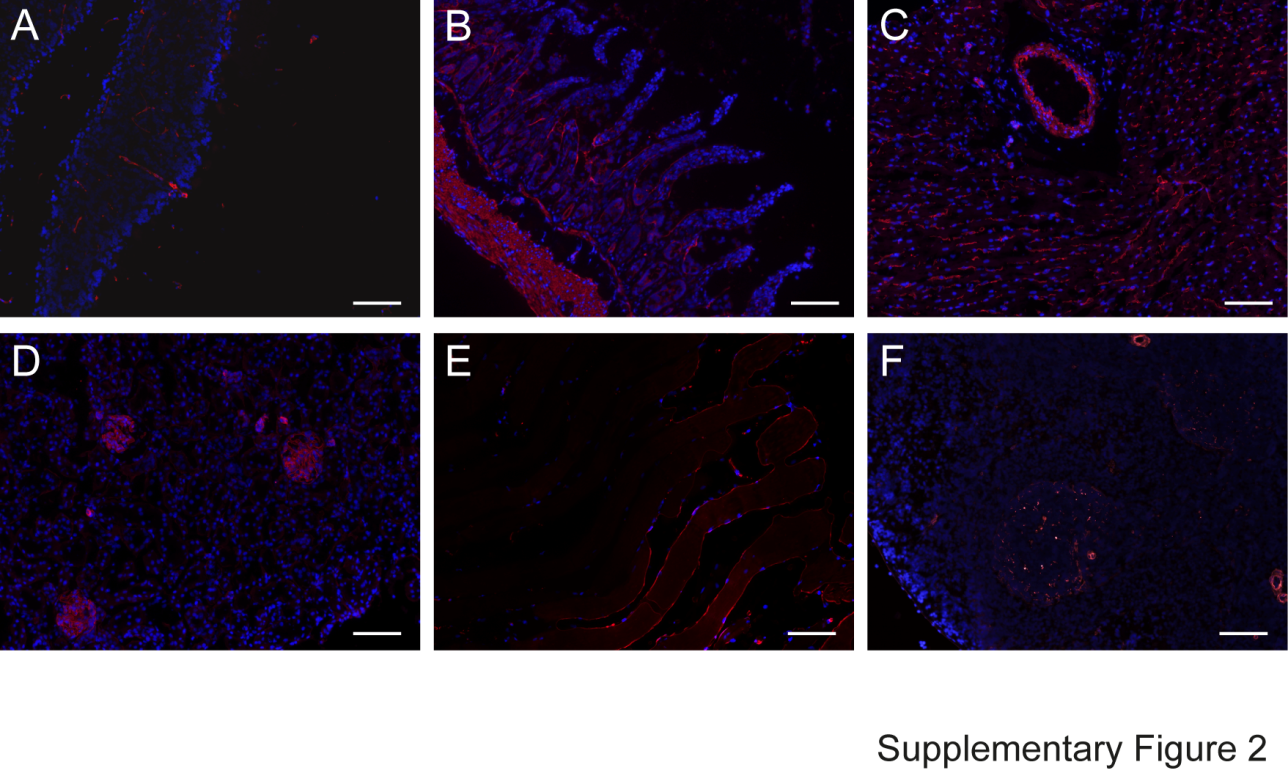


**Fig S2. Representative immunohistochemistry images of laminin α5 in rat tissues.**

(A) Cerebellum. (B) Colon. (C) Myocardium and blood vessel. (D) Kidney. (E) Skeletal muscle. (F) Spleen. The antibody (C13068, Assay Biotechnology) correctly shows immunoreactivity (red) in the epithelial basement membrane of the colon and endothelial basement membranes in cerebellum, colon, spleen, myocardium and kidney, including glomeruli. The antibody also appropriately shows immunoreactivity around cardiomyocytes, skeletal muscle cells and white pulp of the spleen. Nuclei counterstained with DAPI (blue). Images captured with 10x magnification. Scale bar represents 100 µm.
