# Supplementary material for: Characterization of Laminins in Healthy Human Aortic Valves and a Modified Decellularized Rat Scaffold
Source: Biores Open Access. 2020 Dec 7;9(1):269–78. doi: 10.1089/biores.2020.0018 (PMC7757704; doi:10.1089/biores.2020.0018)
Supplement: Supplemental data [file Supp_Table1.docx]

**Supplemental Table 1. Antibodies and immunohistochemistry protocols.**

| *Target* | *Host* | *Target* | *Supplier* | *Clone/Cat. No.* | *Dilution* | *Fixation* | *HIER* | *Blocking Agent* |
| --- | --- | --- | --- | --- | --- | --- | --- | --- |
| Collagen I | Rabbit | Human | Abcam | Ab34710 | 1:100 | Acetone | No | 5% Goat Serum |
| Collagen IV | Rabbit | Rat | Abcam | Ab6586 | 1:200 | Acetone | No | 5% Donkey Serum |
| Elastin | Mouse | Human | Abcam | Clone BA-4 | 1:100 | Acetone | No | 5% Goat Serum |
| Fibronectin | Rabbit | Rat | Abcam | Ab23750 | 1:200 | Acetone | No | 5% Donkey Serum |
| Heparan Sulfate | Mouse | Rat | Amsbio | Clone F58-10E4 | 1:500 | Methanol | No | 1% BSA |
| LAMA1 | Goat | Human | R&D Systems | AF4187 | 1:40 | Acetone | No | 5% Donkey Serum |
| LAMA2 | Mouse | Human | Atlas Antibodies | Clone CL3450 | 1:200 | 4% Formaldehyde/PFA | Yes | 5% Goat Serum |
| LAMA3 | Mouse | Human | Atlas Antibodies | Clone CL3112 | 1:200 | Acetone | Yes | 5% Goat Serum |
| LAMA4 | Mouse | Human/rat | Atlas Antibodies | Clone CL3183 | 1:200 | 4% Formaldehyde/PFA | Yes | 5% Goat Serum |
| LAMA5 | Mouse | Human | Millipore | Clone 4C7 | 1:200 | Metahnol | No | 5% Goat Serum |
| LAMA5 | Rabbit | Rat | Assay Biotechnology | C13068 | 1:200 | Acetone | Yes | 5% Donkey Serum |
| LAMB1 | Mouse | Human | Millipore | Clone 4E10 | 1:100 | Acetone | No | 5% Goat Serum |
| LAMB2 | Mouse | Human | Atlas Antibodies | Clone CL2976 | 1:200 | 4% Formaldehyde/PFA | Yes | 5% Goat Serum |
| LAMB3 | Mouse | Human | Atlas Antibodies | Clone CL3353 | 1:500 | Methanol | Yes | 5% Goat Serum |
| LAMC1 | Mouse | Human | Atlas Antibodies | Clone CL3199 | 1:500 | 4% Formaldehyde/PFA | Yes | 5% Goat Serum |
| LAMC1 | Mouse | Rat | Millipore | Clone 2E8 | 1:200 | Methanol | No | 5% Goat Serum |
| LAMC2 | Mouse | Human | Atlas Antibodies | Clone CL2980 | 1:500 | Acetone | Yes | 5% Goat Serum |
| Perlecan | Mouse | Rat | (N/A)* | Clone 11B4 | 1:100 | Acetone | No | 5% Rabbit Serum |

Gene names used for anti-laminin subunit antibodies for clarity. Target denotes which species the antibody was used for. PFA = paraformaldehyde. HIER = heat-induced epitope retrieval. BSA = bovine serum albumin.

*Not commercially available.
