# Supplementary material for: Characterization of Laminins in Healthy Human Aortic Valves and a Modified Decellularized Rat Scaffold
Source: Biores Open Access. 2020 Dec 7;9(1):269–78. doi: 10.1089/biores.2020.0018 (PMC7757704; doi:10.1089/biores.2020.0018)
Supplement: Supplemental data [file Supp_Fig3.docx]

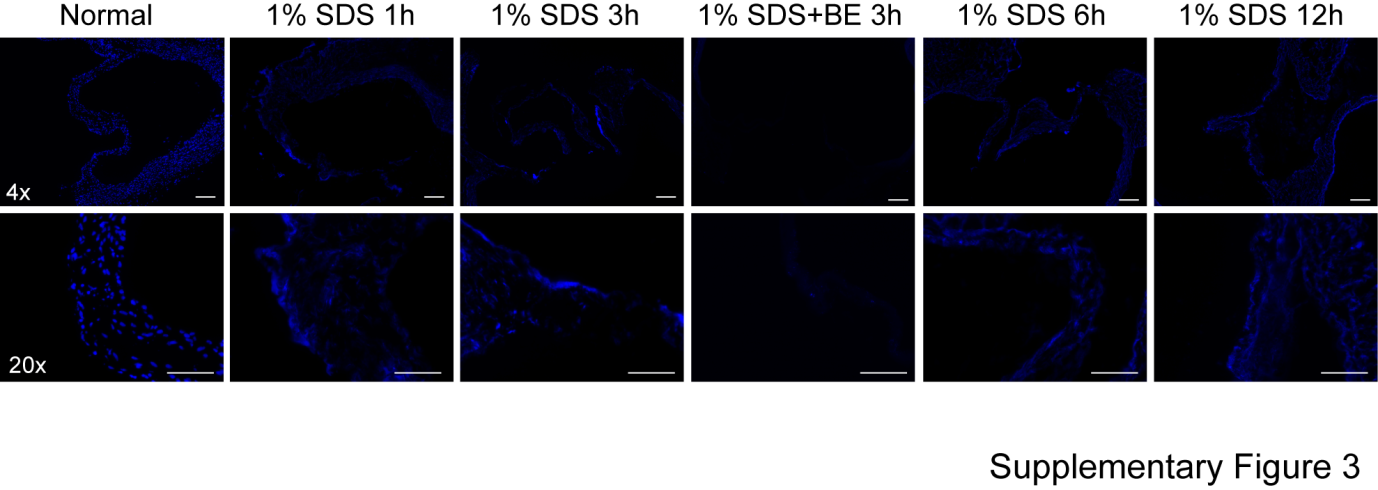


**Fig S3. Representative images of DAPI staining of aortic roots.**

Normal control (n=3) and decellularized with SDS for 1 h (n=1), 3 h (n=1), 6 h (n=1) and 12 h (n=1) and Triton X-100 for 30 min, and SDS for 3 h and Triton X-100 with the addition of benzonase endonuclease treatment. Images were captured at 4x and 20x magnification. SDS = sodium dodecyl sulfate. BE = benzonase endonuclease. Scale bars represent 200 µm (top row) and 100 µm (bottom row).

*Not commercially available.
