# Supplementary material for: Characterization of Laminins in Healthy Human Aortic Valves and a Modified Decellularized Rat Scaffold
Source: Biores Open Access. 2020 Dec 7;9(1):269–78. doi: 10.1089/biores.2020.0018 (PMC7757704; doi:10.1089/biores.2020.0018)
Supplement: Supplemental data [file Supp_Table2.docx]

**Supplemental Table 2. DNA quantification raw data.**

| *Group* | *Specimen* | *DNA Content (ng)* |
| --- | --- | --- |
| Normal controls | 1 | 1123,750 |
|  | 2 | 1011,667 |
|  | 3 | 966,250 |
|  | 4 | 1200,417 |
|  | 5 | 822,917 |
|  | 6 | 1117,500 |
| SDS 3 h | 1 | 331,250 |
|  | 2 | 526,667 |
|  | 3 | 383,333 |
| SDS 3 h + BE | 1 | 76,667 |
|  | 2 | 61,458 |
|  | 3 | 52,083 |
| SDS 12 h | 1 | 518,750 |
|  | 2 | 382,917 |
|  | 3 | 341,667 |

SDS = sodium dodecyl sulfate. BE = benzonase endonuclease.
